# Temperament Development During the First Year of Life in a Sample of Patients with Hearing Impairment Who Participated in the Infants Screening Program in a Single Center in Southern Italy: A Cross-Sectional Study

**DOI:** 10.3390/children12091172

**Published:** 2025-09-02

**Authors:** Carla Laria, Rita Malesci, Antonietta Mallardo, Emma Landolfi, Federica Geremicca D’Ambrosio, Gennaro Auletta, Nicola Serra, Anna Rita Fetoni

**Affiliations:** Institute of Audiology, Department of Neurosciences, Reproductive Sciences and Dentistry, University of Naples Federico II, 80138 Naples, Italy; carla.laria@unina.it (C.L.); antoniet.mallardo@studenti.unina.it (A.M.); emyla@hotmail.com (E.L.); federicageremicca@gmail.com (F.G.D.); auletta@unina.it (G.A.); nicola.serra@gmail.com (N.S.); annarita.fetoni@unina.it (A.R.F.)

**Keywords:** infants, QUIT questionnaire, early auditory deprivation, temperament, socio-emotional impairment

## Abstract

**Highlights:**

**What are the main findings?**

- The development of temperament in the newborn and its interaction with listening skills is a complex process and its evaluation requires a longer observation time for the child than a year of life.

**What is the implication of the main finding?**

- The QUIT questionnaire proved to be a more effective tool for raising parents’ awareness of children’s behavioral and cognitive problems than for drawing a behavioral profile.
- This study suggests the need for a multidisciplinary approach in the evaluation of newborns with hearing loss.

**Abstract:**

Background/Objectives: Temperament is an innate personality trait, influenced by genetic, biological, and environmental factors. Hearing loss, particularly during the critical period of auditory development, can influence cognitive and temperamental development. This study aims to assess the impact of hearing loss on temperamental development in infants aged between 1 and 12 months. Methods: A cross-sectional study of a sample of 132 pediatric patients from the infant hearing screening program was conducted from June 2023 to June 2024. The infants were divided into two groups based on hearing status and the presence of risk factors; cognitive and temperamental parameters were assessed using the QUIT questionnaire. Results: No significant differences were found between infants with and without hearing loss when also considering the infants without risk factors. Normal temperament was found in infants with and without hearing loss, considering both risk and non-risk factors. Finally, no relationship between hearing loss degree and temperament type, considering both the absence and presence of risk factors, was observed. Conclusions: In the early months of an infant’s life, hearing loss does not appear to significantly affect temperamental development. Only through the monitoring of these hearing-impaired infants to detect more severe hearing loss and/or in the presence of other risk factors can deviant development be hypothesized. In this regard, multidisciplinary evaluations may be crucial for the early detection and correction of dysfunctional behaviors.

## 1. Introduction

Temperament is a set of behavioral patterns with interindividual variability, which are based on the innate characteristics of an individual, including biological, neurophysiological, and constitutional (genetic and perinatal) factors, which are observable through the frequency and intensity of reactions to different environmental stimuli, and which also include emotional aspects of personality [1]. Such differences emerge very early, not just from the very first months of life, but during intrauterine life. The temperament profile appears relatively stable over time and in different situations; however, it can be changeable, modulated by different environmental conditions and the child’s life experience [2].

Taking into account emotional responses, four temperamental profiles can be defined [3]: Emotional Temperament, with high emotional reactivity, both positive and negative (child usually described as lively); Calm Temperament, with low emotional reactivity, both positive and negative (child who smiles little); Normal Temperament, with positive reactivity greater than negative reactivity (child is sunny and positive); Difficult Temperament, with negative reactivity greater than positive reactivity (child is easily irritable, tends to be sad or defined as intractable).

It is important to recognize the different temperamental profiles as early as possible as, in some cases, they may be a tell-tale sign of a neuropsychiatric disorder that will manifest as the child grows up, such as ADHD, anxiety, or depression [3].

Hearing loss, especially during the first two years of life, coincides with the period when the brain is highly plastic. During this period, a lack of auditory stimuli can trigger compensatory neural reorganization in areas of the brain typically involved in the auditory process, such as the prefrontal cortex and temporal lobe. This reorganization could be at the expense of other cognitive functions, including attention, working memory, and executive function [4].

Hearing impairment increases the likelihood of the development of dysfunctional behaviors and socio-emotional difficulties [5]. Thus, deafness can prevent children from establishing valid communication relationships, especially in the dyadic mother–child relationship, due to the difficulty of maintaining joint linguistic attention and the cognitive prerequisites needed for the development of temperament, and these executive functions (such as working memory and inhibitory control) are fundamental for the development of language and academic success in later years [6,7].

Previous studies [8,9] confirm a link between temperament and language and allow us to highlight a relationship between temperamental profiles and languages with different characteristics and levels of competence (particularly between positive emotionality/attention and lexical ability): increased word production correlates with higher levels of positive emotionality and attention.

Early auditory rehabilitation is essential for the development of hearing and perception skills in infants with hearing loss. Indeed, it is important to monitor the development of cognitive, emotional, and motor skills, especially in infants with severe or profound hearing loss, to provide a rehabilitation plan tailored to each child’s specific needs to support their cognitive, emotional, and temperamental development.

Objectives: This study aims to assess the impact of hearing loss on temperamental development in infants aged between 1 and 12 months.

## 2. Materials and Methods

### 2.1. Study Design and Population

A cross-sectional study was conducted according to the STROBE checklist for cross-sectional studies (https://www.strobe-statement.org/download/strobe-checklist-cross-sectional-studies-pdf; last access: 1 August 2025) from June 2023 to June 2024, with a sample of 132 pediatric patients pertaining to the neonatal hearing screening pathway at the Audiology and Phoniatrics Unit of the University of Naples Federico II. The selected infants were aged between 1 and 12 months.

Based on the presence or absence of risk factors (RF) for hearing loss, the sample was stratified into the following groups:

Group 1: infants with normal hearing; Group 2: infants with hearing loss; Group 1A: infants with normal hearing and without risk factors; Group 2A: infants with hearing loss and without risk factors; Group 1B: infants with normal hearing and risk factors; Group 2B: infants with hearing loss and risk factors.

Specifically, the risk factors considered were (1) hospitalization in TIN for a period of five days or more, (2) infections of the TORCH complex, (3) familiarity with hearing loss, and (4) presence of genetic syndrome. In this way, it was possible to separately evaluate the influence of hearing loss and risk factors on temperament.

**Figure 1:**
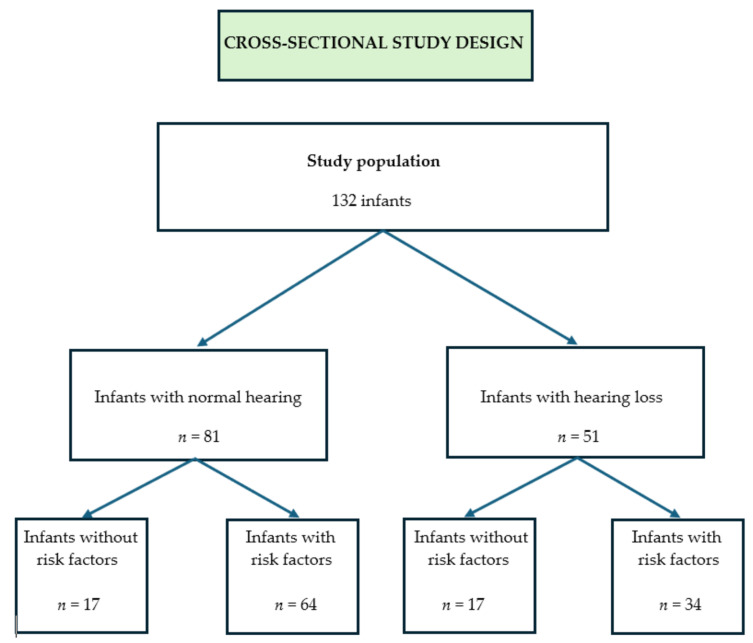
Flowchart of our study.

In Figure 1, we present the flowchart of our study.

### 2.2. Inclusion and Esclusion Criteria

All pediatric patients aged between one and twelve months involved in a neonatal hearing screening were enrolled.

### 2.3. Audiological and Phonology Evaluation

All infants included in the study underwent a clinical and instrumental audiological evaluation, with impedance-metric examination and auditory-evoked brainstem (ABR) potentials for an assessment of auditory function. The ABR diagnostic assessment with threshold identification was performed by a specialist audiologist in our pediatric audiology service, in a soundproofed and faradized room, during spontaneous sleep. The device used was Neuro-Audio, Inventis. The test was performed with standard skin preparation and three assembly electrodes with impedance maintained at 3000 dine. An active electrode was applied to the forehead, and an explorer electrode was placed on the omolateral mastoid; one was a counter-lateral mass electrode. The standard procedure consists of alternating clicks at 21 pps, with a duration of 0.1 ms, 100–2000 Hz filter settings, and an analysis time of 12 ms. The protocol begins with a monaural stimulation at 80 dB HL for the identification of the three main waves—I, III and, V—for peak determination and interpeak latencies. After this step, the stimulus is reduced by 10 dB to a minimum of 20 dB HL. Normal hearing was defined based on the persistent presence of V waves for acoustic stimuli <30 dB nHL, and HL was defined as the persistent presence of V waves for acoustic stimuli 30 dB nHL. In hearing-impaired people, the classification of hearing loss degree is based on the BIAP (International Bureau for Audiophonology—1996) [10] protocol and includes normal (<20 dB HL), mild (21–40 dB HL), moderate (41–70 dB HL), severe (71–90 dB HL), and profound (>91 dB HL). Finally, infants with unilateral hearing loss were included in the mild hearing loss group, according to Tharpe [11].

### 2.4. Instrument and Administration

In this study, for the evaluation of temperamental characteristics in infants, the QUIT questionnaire was used. The QUIT validation questionnaire was presented according to the authors’ guidelines and administered before the audiological examination performed with the ABR to avoid communication of the diagnosis to the parents influencing their assessment of the infant’s temperament. The QUIT questionnaire was found to be the most appropriate instrument for the study groups considered in the assessment of temperament in infants aged 1 to 12 months.

The QUIT questionnaire is characterized by six dimensions: Social Orientation, Novelty Inhibition, Motor Activity, Positive Emotionality, Negative Emotionality, and Attention. There are four types of QUIT questionnaires that use the same theoretical construct to measure temperament in four different age groups; in this study, we considered the one regarding the psychometric characteristics of infants aged 1–12 months.

The parents of the infants were invited to provide information about the family and infant care, and to answer the questions of the QUIT Questionnaire (Italian Temperament Questionnaire) [3].

There are four versions: 1 to 12 months, 13 to 36 months, 3 to 6 years, and 8 to 11 years. Only the version with patients between 1 and 12 months was considered in the study. The questionnaire can be filled in by any figure who spends many hours with the baby (including relatives, teachers, and educators) and can produce a high test reliability index score. The QUIT 1–12 months program provides for the evaluation of 55 items, divided into three categories referring to three situational frameworks: (i) the child with others (eye contact, screaming/crying/smiling/indifference, level of motor agitation in different situations with the mother or other people); (ii) the child playing (exploration of objects, amplitude of movements performed, attention/distractibility, emotional display); (iii) the child in front of the news (with unknown people, animals or objects, in new environments, after hearing unfamiliar noises).

The caregivers were given plenty of time to think about the baby’s behavior over the weeks before completing the questionnaire and to provide a reliable response. The purpose of the questionnaire is to assess the six temperament dimensions: social orientation, inhibition of novelty, motor activity, positive emotional, negative emotions, attention. Based on the results obtained, it was possible to distinguish between “positive” or “problematic” types of adaptation. Specifically, by comparing the results regarding the child’s emotionality (positive and negative), it was possible to identify the following temperament profiles, as shown in Table 1:

- Emotional temperament, with high emotional responsiveness, both positive and negative (child usually described as lively).
- Calm temperament, with low emotional responsiveness, both positive and negative (child who smiles little).
- Normal temperament, with greater positive reactivity than negative reactivity (sunny and positive child).
- Difficult temperament, with a negative reactivity greater than the positive one (child easily irritable, mostly sad or defined as unmanageable).

In Table 1, we summarize the temperamental profiles obtained with the QUIT questionnaire.

These profiles are important to recognize, because in some cases they can be a sign of a neuropsychiatric disorder that will develop with growth, such as ADHD, anxiety, or depression [4].

### 2.5. Statistical Analysis

Data are presented as a number and percentage for categorical variables, and continuous data are expressed as the mean ± standard deviation (SD) unless otherwise specified. The chi-square test and Fisher’s exact test were performed to evaluate significant differences in proportions or percentages between the two groups. The multiple comparison chi-square and Fisher’s exact test were used to define significant differences among three or more percentages for unpaired data. If the chi-square or Fisher’s exact test were significant (*p*-value < 0.05), then a post hoc test was performed using the Adjusted Standardized Residuals and the Z-test. Fisher’s exact test was used where the chi-square test was not appropriate. The test for normal distribution was performed using the Shapiro–Wilk test. The *t*-test was used to test the differences between two means of unpaired data. Alternatively, the Mann–Whitney test was used if the distributions were not normal. Particularly, where the tests on medians showed a significant difference and the medians were equal, then the mean rank values were described. In addition, the chi-square goodness of fit was used to evaluate significant differences among three or more mutually exclusive modalities of a variable. One-way analysis of variance was used to test the difference between the means of several independent subgroups. If the ANOVA test was positive (*p*-value < 0.05), then Scheffé’s post hoc test for the pairwise comparison of subgroups was performed. Alternatively, the Kruskal–Wallis test (H-test) was used to analyze the effect of a classification factor on ordinal data if the distributions were not normal. Finally, all tests with *p*-value (*p*) < 0.05 were considered significant. The statistical analysis was performed using the Matrix Laboratory (MATLAB) analytical toolbox version 2008 (MathWorks, Natick, MA, USA). for Windows with 32 bits.

## 3. Results

The statistical analyses were performed on a sample of 132 consecutive pediatric patients comprising 62.1% males and 37.9% females, with age at admission in the range 1–12 months, with a mean of 5.4 months and standard deviation of 3.9.

Fifty-one infants showed hearing loss: 27.5% (14) had unilateral hearing loss (left or right), while 72.5% (37) had bilateral hearing loss. Firstly, we evaluated the type of hearing loss: 82.4% (42) of infants had sensorineural hearing loss, 11.8% (6) a transmissive hearing loss, and 5.9% (3) a mixed form. Regarding the degree of hearing loss, 24.5% (12) of infants had a mild form, 39.2% (20) had a moderate form, 15.7% (8) had a severe form, and 21.6% (11) had a profound form.

In Table 2, we describe our total sample and its stratification into two groups, i.e., infants with and without hearing loss. In the last column, we provide a comparison between the two groups.

As shown in Table 2, significant relationships were observed between hearing loss and family history and hearing loss and intensive care. Particularly, we found more infants with family history in Group 2 as compared to Group 1 (19.6% vs. 2.5%, *p* = 0.0013); however, we found that permanent presence in intensive care was a more common risk factor in the Group 1 infants compared to Group 2 (58.0% vs. 25.5%, *p* = 0.0003).

Table 3 shows the parameters of the QUIT questionnaire for Groups 1 and 2. In the last column, we present the comparison between the two groups.

In Table 3, focusing on QUIT dimensions, no significant differences were observed between infants with and without hearing loss. Table 4 shows the sample stratified in infants without (Group 1A) and with hearing loss (Group 2A), considering only infants without risk factors.

In Table 4, we found no significant differences between infants with and without hearing loss.

In Table 5, we reported the parameters of QUIT questionnaire, comparing infants with and without hearing loss and considering only infants without risk factors.

Regarding cognitive parameters, no significant differences between infants with and without hearing loss were observed when considering only infants without risk factors.

In Table 6 and Table 7, we report on a stratified sample of infants with and without hearing loss, including risk factors, to evaluate the impact of risk factors.

In Table 6, we found, in infants with risk factors, a significant presence of hypoacoustic familiarity (29.4%, *p* = 0.0003) in infants with hearing loss, with a stronger presence observed in infants in intensive care with no hearing loss (71.9%, *p* = 0.0012).

Table 7 shows no significant differences in temperament between infants with no hearing loss and hearing loss when considering the risk factors

**Figure 2:**
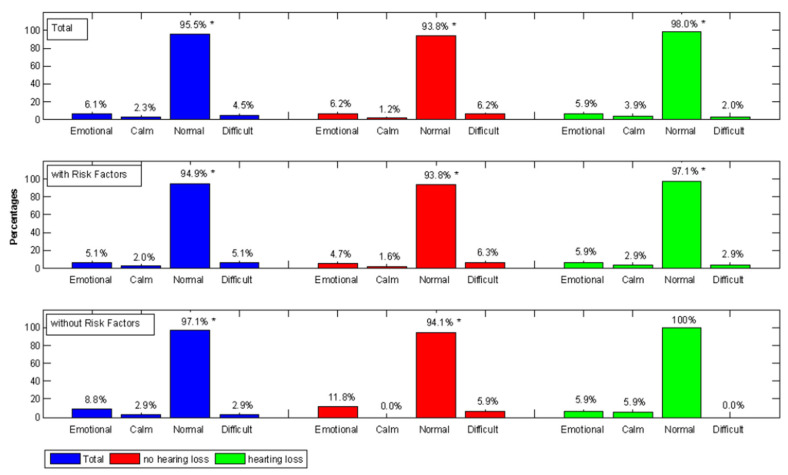
Temperament type of infants with and without hearing loss, considering infants with and without risk factors. The asterisk represents a significant percentage.

In Figure 2, we present the temperaments evaluated using the QUIT questionnaire, considering the total sample, in infants with and without hearing loss.

Figure 2 shows a significant presence of normal infants for each group. In Table 8, we present the possible relationship between temperament type and hearing loss degree.

In Table 8, we can observe that there is no relationship between hearing loss degree and temperament type considering both the absence and presence of risk factors.

## 4. Discussion

This study examined the relationship between hearing loss and temperament development in a cohort of 1–12-month-old infants participating in the neonatal hearing screening program at the Audiology and Phoniatrics Unit of the A.O.U. Federico II of Naples. Our results do not show a significant correlation between hearing loss and the temperamental profile of infants.

### 4.1. Influence of Parents’ Work and Education Level in the Evaluation of the Infant Temperament Using the QUIT Questionnaire

Comparing data between infants with and without hearing loss, we observed no significant differences in terms of parental type of work and education level, i.e., parents of both groups performed a temperament assessment using the QUIT questionnaire with the same sociodemographic conditions. The same results were observed for infants with and without hearing loss, and with and without risk factors.

### 4.2. Influence of the Infant’s Age and Degree of Hearing Loss on Temperament

This study evaluated the development of temperament in deaf infants compared to the degree of hearing loss. Surprisingly, no differences were found in the temperamental development of infants with hearing loss compared to normal hearing. Moreover, even in the group of infants with hearing loss, no statistically significant differences were found in relation to the severity of the disorder.

Few studies have focused on this field. Our results are in contrast with Castellanos et al. [12], who found significant differences in temperament between children with and without pre-language hearing loss through the administration of the Early Childhood Behavior Questionnaire (ECBQ), a tool used to assess temperament between 18 and 36 months.

Our results could be due to the young age of the children, between 1 and 12 months, which represents a real obstacle to the correct interpretation of their behavior, and to the use of a different questionnaire that includes more age groups. Therefore, some assessments could not be interpreted very accurately because of the children’s age was too young, and their behaviors were difficult to decipher by both parents and health care workers, except in cases where obvious difficulties were evident. Therefore, we hypothesize that the QUIT questionnaire should be used with caution in infants between 1 and 12 months.

### 4.3. Influence of Risk Factors on Temperament

An important aspect of this study was the stratification of infants into subgroups based on the presence of additional risk factors, such as prenatal infections, genetic syndromes, or a family history of hearing loss.

In our study, similar temperament profiles were found among normal hearing or hearing-impaired children, with or without additional risk factors, as if the risk factors that were examined were not an aggravating condition for the development of a more deviant risk profile, at least in the early stages of life.

This is in disagreement with the studies found in the international literature. For example, Montirosso et al. [13], using the QUIT questionnaire and the Beck Depression Inventory (BDI), and focusing on children in the 6–12 months age group, found a marked difference in the development of temperament between high- and low-risk premature infants and term infants. Other studies on children with normal hearing exposed to risk factors have shown that this population is more fragile than those not exposed in the development of possible delays at various stages of life [14,15].

Remarkably, Caravale et al. [16] suggested a possible correlation between sleep disturbance and temperament in a sample of premature and born-to-term children of 2 years old. Premature infants showed more frequent sleep difficulties and restlessness than full-term children, with lower scores on the QUIT questionnaire in terms of attention and social orientation, and higher scores in terms of negative emotional and motor activity. These results suggest how gestational age affects temperament profile; specifically, high-risk children have more difficulties in social adaptation than low-risk children. In our data, the early evaluation of infants affected by hearing loss, with or without risk factors, suggests that a longitudinal follow-up should be undertaken, even in children with an apparently normal temperament profile, and particularly in deaf infants with associated risk factors. In fact, infants can later present with a more unstable temperament profile (emotional/difficult); therefore, early and specialized intervention is required. When mainly considering the young age of the children, the QUIT questionnaire may be considered less sensitive when evaluating specific aspects of the child’s development; thus, more information could be provided in later developmental periods.

A longitudinal evaluation of these children may be useful to provide indicators of the development of temperament, especially in children with hearing loss, co-morbidities, and other risk factors [17].

Furthermore, the QUIT questionnaire can be considered a good measurement tool, but we suggest using it together with other scales in infants between 1 and 12 months.

### 4.4. Limitations

The results obtained should be confirmed on a larger sample. However, this does not take away the fact that the use of adequate statistical tests for small samples reduced the possibility of statistical biases in the results obtained to a minimum.

A further limitation of the study is the administration of QUIT, as well as all questionnaires administered by parents, which depend on the subjective ability of parents to assess the infant’s temperament, such as the ability of the family to observe and analyze the behavior of infants in different stimulation contexts and also their ability to correctly interpret the skills that the clinician wishes to investigate, as well as the psychological condition of the parents at the time of administration of the questionnaire.

## 5. Conclusions

Although some standardized assessment tools have been proposed to measure temperament, they may not be entirely useful for understanding the interactions between cognitive development and auditory processing. However, QUIT can provide a useful tool for raising parents’ awareness of children’s behavioral and cognitive problems. Future studies in a larger cohort will provide evidence of the temperamental profile of younger deaf children. Overall, this study strongly suggests the need for a comprehensive, multidisciplinary approach to the evaluation and management of infants with hearing loss. 

## Figures and Tables

**Table 1:** Temperamental profiles in relation to the correlation between positive and negative emotionality.

Temperamental Profile	Positive Emotionality	Negative Emotionality
emotional	↑	↑
calm	↓	↓
normal	↑	↓
difficult	↓	↑

↑ = positive; ↓ = negative.

**Table 2:** Comparison between infants with and without hearing loss, including infants with risk factors, and considering all socio-demographic parameters. Sample size was reported if different from the total.

Parameters	Total	Group 1: No Hearing Loss	Group 2: Hearing Loss	Group 1 vs. Group 2 *p*-Value (Test)
*Infants*	132	61.4% (81)	38.6% (51)	
*Age at admission*				
Mean ± SD	5.4 ± 3.9	4.5 ± 2.0	5.5 ± 3.5	
Median (IQR)	4.0 (3.0, 6.0)	4.0 (3.0, 5.0)	5.0 (3.0, 7.0)	0.35 (MW)
*Gender*				
Male	62.1% (82)	61.7% (50)	62.7% (32)	0.91 (C)
Female	37.9% (50)	38.3% (31)	37.3% (19)	
*Infants with risk factors*	74.2% (98)	79.0% (64)	66.7% (34)	0.11 (C)
*Prenatal infection*	19.7% (26)	23.5% (19)	13.7% (7)	0.17 (C)
*Ear malformation*	3.8% (5)	2.5% (2)	5.9% (3)	0.37 (F)
*Genetic syndrome*	3.8% (5)	2.5% (2)	5.9% (3)	0.37 (F)
*Hypoacoustic familiarity*	9.1% (12)	2.5% (2)	19.6% (10)	0.0013 * (F)
*Intensive care*	45.5% (60)	58.0% (47)	25.5% (13)	0.0003 * (C)
*Days of intensive care*		*n* = 47	*n* = 13	
Mean ± SD	22.4 ± 19.5	21.7 ± 17.2	25.2 ± 27.2	
Median (IQR)	16.5 (7.0, 30.0)	18.0 (8.25, 29.5)	15.0 (5.75, 40.25)	0.61 (MW)
*Parents*				
*Family members*		*n* = 80	*n* = 51	
Mean ± SD	1.6 ± 0.8	1.6 ± 0.8	1.6 ± 0.8	
Median (IQR)	2.0 (1.0, 2.0)	2.0 (1.0, 2.0)	2.0 (1.0, 2.0)	
Mean rank		66.4	65.3	0.85 (MW)
*Mother’s profession*				
No response	0.8% (1)	1.2% (1)	0.0% (0)	
Unemployed	52.3% (69)	50.6% (41)	54.9% (28)	
Student	1.5% (2)	1.2% (1)	2.0% (1)	0.41 (F)
Housewife	3.0% (4)	3.7% (3)	2.0% (1)	
Freelance	15.9% (21)	12.3% (10)	21.6% (11)	
Employee	26.5% (35)	30.9% (25)	19.6% (10)	
*Father’s profession*				
No response	3.0% (4)	4.9% (4)	0.0% (0)	
Unemployed	10.6% (14)	7.4% (6)	15.7% (8)	
Freelance	24.2% (32)	23.5% (19)	25.5% (13)	0.35 (C)
Employee	62.1% (82)	64.2% (52)	58.8% (30)	
*Mother’s education level*				
No response	0.8% (1)	1.2% (1)	0.0% (0)	
Primary school diploma	4.5% (6)	3.7% (3)	5.9% (3)	
Middle school diploma	30.3% (40)	27.2% (22)	35.3% (18)	0.63 (F)
High school diploma	34.1% (45)	34.6% (28)	33.3% (17)	
Bachelor’s degree	30.3% (40)	33.3% (27)	25.5% (13)	
*Father’s education level*				
No response	3.0% (4)	4.9% (4)	0.0% (0)	
Primary school diploma	3.0% (4)	4.9% (4)	0.0% (0)	
Middle school diploma	24.2% (32)	23.5% (19)	25.5% (13)	0.52 (F)
High school diploma	53.0% (70)	50.6% (41)	56.9% (29)	
Bachelor’s degree	16.7% (22)	16.0% (13)	17.6% (9)	
*Care by grandparents*	15.2% (20)	17.3% (14)	11.8% (6)	0.74 (C)
*Care by parents*	99.2% (131)	98.8% (80)	100% (51)	1.0 (F)

SD = standard deviation; IQR = interquartile interval; * = significant test; C = chi-square test; MW = Mann–Whitney test; F = Fisher’s exact test. The modality “no response” was excluded from all analyses.

**Table 3:** Comparison between infants with and without hearing loss considering all QUIT dimensions.

QUIT Questionnaire				
Parameters	Total	Group 1: No Hearing Loss	Group 2: Hearing Loss	Group 1 vs. Group 2 *p*-Value (Test)
*Infants*	132	38.6% (51)	61.4% (81)	
*Social orientation score*				
Mean ± SD	4.7 ± 0.8	4.8 ± 0.7	4.6 ± 0.8	0.16 (T)
Median (IQR)	4.8 (4.3, 5.3)	4.9 (4.4, 5.3)	4.7 (4.1, 5.3)	
*Novelty Inhibition score*		*n* = 77	*n* = 50	
Mean ± SD	2.1 ± 0.7	2.1 ± 0.8	2.1 ± 0.7	
Median (IQR)	2.1 (1.5, 2.6)	2.1 (1.5, 2.6)	2.0 (1.6, 2.5)	0.63 (MW)
*Motor Activity score*				
Mean ± SD	3.9 ± 0.9	3.8 ± 0.9	4.0 ± 0.9	0.73 (T)
Median (IQR)	3.8 (3.2, 4.5)	3.7 (3.2, 4.3)	3.9 (3.2, 4.6)	
*Positive Emotionality score*				
Mean ± SD	5.0 ± 0.9	5.0 ± 0.9	5.0 ± 0.9	
Median (IQR)	5.1 (4.6, 5.7)	5.1 (4.6, 5.6)	5.2 (4.5, 5.7)	0.96 (MW)
*Negative Emotionality score*				
Mean ± SD	2.6 ± 0.8	2.7 ± 0.7	2.6 ± 0.8	
Median (IQR)	2.6 (2.1, 3.0)	2.6 (2.1, 3.0)	2.5 (2.0, 3.1)	0.47 (MW)
*Attention score*	*n* = 131	*n* = 80	*n* = 51	
Mean ± SD	4.5 ± 0.8	4.6 ± 0.9	4.4 ± 0.8	
Median (IQR)	4.6 (4.1, 5.1)	4.6 (4.2, 5.2)	4.6 (4.0, 5.0)	0.26 (MW)

SD = standard deviation; IQR = interquartile interval; T = *t*-test; MW = Mann–Whitney test.

**Table 4:** Socio-demographic characteristics of parents and infants without and with hearing loss, and without risk factors. In each row, we report the sample size if different from the total.

Parameters	Group 1A: No Hearing Loss and Without Risk Factors	Group 2A: Hearing Loss and Without Risk Factors	Group 1A vs. Group 2A *p*-Value (Test)
*Infants*	21.0% (17/81)	33.3% (17/51)	
*Age at admission*			
Mean ± SD	4.6 ± 3.2	5.1 ± 4.8	
Median (IQR)	4.0 (2.0, 6.5)	3.0 (2.75, 6.25)	0.92 (MW)
*Gender*			
Male	47.1% (8)	64.7% (11)	0.30 (C)
Female	52.9% (9)	35.3% (6)	
*Parents*			
*Family members*			
Mean ± SD	1.5 ± 0.8	1.5 ± 0.8	
Median (IQR)	1.0 (1.0, 2.0)	1.0 (1.0, 2.0)	
Mean rank	17.5	17.5	1.0 (MW)
*Mother’s profession*			
No response	0.0% (0)	0.0% (0)	
Unemployed	47.1% (8)	35.3% (6)	
Student	5.9% (1)	0.0% (0)	0.62 (F)
Housewife	5.9% (1)	5.9% (1)	
Freelance	17.6% (3)	41.2% (7)	
Employee	23.5% (4)	17.6% (3)	
*Father’s profession*			
No response	5.9% (1)	0.0% (0)	
Unemployed	5.9% (1)	11.8% (2)	1.0 (F)
Freelance	29.4% (5)	35.3% (6)	
Employee	58.8% (10)	52.9% (9)	
*Mother’s education level*			
No response	0.0% (0)	0.0% (0)	
Primary school diploma	5.9% (1)	5.9% (1)	0.95 (F)
Middle school diploma	29.4% (5)	29.4% (5)	
High school diploma	41.2% (7)	29.4% (5)	
Bachelor’s degree	23.5% (4)	35.3% (6)	
*Father’s education level*			
No response	5.9% (1)	0.0% (0)	
Primary school diploma	0.0% (0)	0.0% (0)	0.89 (F)
Middle school diploma	29.4% (5)	23.5% (4)	
High school diploma	47.1% (8)	58.8% (10)	
Bachelor’s degree	17.6% (3)	17.6% (3)	
*Care by grandparents*	17.6% (3)	5.9% (1)	0.60 (F)
*Care by parents*	100% (17)	100% (17)	1.0 (F)

SD = standard deviation; IQR = interquartile interval; C = chi-square test; MW = Mann–Whitney test; F = Fisher’s exact test. The modality “no response” was excluded from all analyses.

**Table 5:** Temperament dimensions of infants with and without hearing loss, and without risk factors. In each row, we report the sample size if different from the total.

QUIT Questionnaire			
Temperament Dimensions	Group 1A: No Hearing Loss and Without Risk Factors	Group 2A: Hearing Loss and Without Risk Factors	Group 1A vs. Group 2A *p*-Value (Test)
*Infants*	21.0% (17/81)	33.3% (17/51)	
*Social orientation score*			
Mean ± SD	4.9 ± 0.9	4.8 ± 1.0	0.93 (T)
Median (IQR)	5.1 (4.5, 5.5)	4.9 (3.8, 5.5)	
*Novelty Inhibition score*	*n* = 16	*n* = 17	
Mean ± SD	2.0 ± 0.6	2.0 ± 0.5	0.96 (T)
Median (IQR)	1.8 (1.3, 2.5)	2.0 (1.6, 2.3)	
*Motor Activity score*			
Mean ± SD	3.7 ± 1.0	3.6 ± 0.8	0.58 (T)
Median (IQR)	3.7 (3.2, 4.0)	3.4 (2.9, 4.1)	
*Positive Emotionality score*			
Mean ± SD	4.9± 1.2	5.2 ± 1.0	0.61 (T)
Median (IQR)	5.1 (4.9, 5.6)	5.4 (4.6, 5.7)	
*Negative Emotionality score*			
Mean ± SD	2.8 ± 0.7	2.5 ± 0.8	
Median (IQR)	2.7 (2.4, 2.9)	2.6 (1.8, 2.8)	0.25 (MW)
*Attention score*			
Mean ± SD	4.4 ± 1.1	4.5 ± 0.8	
Median (IQR)	4.4 (4.1, 5.3)	4.4 (4.1, 5.3)	
Mean rank	17.5	17.5	1.0 (MW)

SD = standard deviation; IQR = interquartile interval; T = *t*-test; MW = Mann–Whitney test.

**Table 6:** Socio-demographic characteristics of parents and infants without and with hearing loss, including risk factors (in each row, we report the sample size if different from the total).

Parameters	Group 1B No Hearing Loss and Risk Factors	Group 2B Hearing Loss and Risk Factors	Group 1B vs. Group 2B *p*-Value (Test)
*Infants*	79.0% (64/81)	66.7% (34/51)	
*Age at admission*			
Mean ± SD	4.5 ± 1.6	5.7 ± 2.7	
Median (IQR)	4.0 (4.0, 5.0)	5.0 (3.0, 7.0)	0.07 (MW)
*Gender*			
Male	34.4% (42)	61.8% (21)	0.14 (C)
Female	65.6% (22)	38.2% (13)	
*Prenatal infection*	29.7% (19)	20.6% (7)	0.94 (C)
*Ear malformation*	3.1% (2)	8.8% (3)	0.34 (F)
*Genetic syndrome*	3.1% (2)	8.8% (3)	0.34 (F)
*Hypoacoustic familiarity*	3.1% (2)	29.4% (10)	0.0003 * (F)
*Intensive care*	71.9% (46)	38.2% (13)	0.0012 * (C)
*Days of intensive care*	*n* = 46	*n* = 13	
Mean ± SD	22.1 ± 17.1	25.2 ± 27.2	
Median (IQR)	19.0 (9.0,30.0)	15.0 (5.75, 40.25)	0.53 (MW)
*Parents*			
*Family members*			
Mean ± SD	1.7 ± 0.8	1.6 ± 0.8	
Median (IQR)	2.0 (1.0, 2.0)	2.0 (1.0, 2.0)	
Mean rank	49.2	48.6	0.91 (MW)
*Mother’s profession*			
No response	1.6% (1)	0.0% (0)	
Unemployed	51.6% (33)	64.7% (22)	0.35 (F)
Student	0.0% (0)	2.9% (1)	
Housewife	3.1% (2)	0.0% (0)	
Freelance	10.9% (7)	11.8% (4)	
Employee	32.8% (21)	20.6% (7)	
*Father’s profession*			
No response	4.7% (3)	0.0% (0)	
Unemployed	7.8% (5)	17.6% (6)	0.38 (F)
Freelance	21.9% (14)	20.6% (7)	
Employee	65.6% (42)	61.8% (21)	
*Mother’s education level*			
No response	1.6% (1)	0.0% (0)	
Primary school diploma	3.1% (2)	5.9% (2)	0.48 (F)
Middle school diploma	26.6% (17)	38.2% (13)	
High school diploma	32.8% (21)	32.4% (11)	
Bachelor’s degree	35.9% (23)	23.5% (8)	
*Father’s education level*			
No response	4.7% (3)	0.0% (0)	
Primary school diploma	6.2% (4)	0.0% (0)	0.59 (F)
Middle school diploma	21.9% (14)	25.6% (9)	
High school diploma	51.6% (33)	55.9% (19)	
Bachelor’s degree	15.6% (10)	17.6% (6)	
*Care by grandparents*	17.2% (11)	14.7% (5)	0.75 (C)
*Care by parents*	98.4% (63)	100% (34)	1.0 (F)

SD = standard deviation; IQR = interquartile interval; * = significant test; C = chi-square test; MW = Mann–Whitney test; F = Fisher’s exact test. The modality “no response” was excluded from all analyses.

**Table 7:** Temperament dimensions of infants with and without hearing loss, including risk factors. In each row, we report the sample size if different from the total.

Temperament Dimensions	Group 1B No Hearing Loss and Risk Factors	Group 2B Hearing Loss and Risk Factors	Group 1B vs. Group 2B *p*-Value (Test)
*Social orientation score*			
Mean ± SD	4.8 ± 0.7	4.5 ± 0.7	0.054 (T)
Median (IQR)	4.9 (4.4, 5.3)	4.7 (4.2, 5.0)	
*Novelty Inhibition score*	*n* = 61	*n* = 33	
Mean ± SD	2.2 ± 0.8	2.1 ± 0.8	0.86 (T)
Median (IQR)	2.2 (1.6, 2.6)	2.0 (1.6, 2.6)	
*Motor Activity score*			
*Mean ± SD*	3.8 ± 0.9	4.2 ± 0.9	0.054 (T)
*Median (IQR)*	3.8 (3.2, 4.3)	4.3 (3.7, 4.7)	
*Positive Emotionality score*			
*Mean ± SD*	5.0 ± 0.9	4.9 ± 0.9	
*Median (IQR)*	5.1 (4.6, 5.7)	5.1 (4.3, 5.6)	0.72 (MW)
*Negative Emotionality score*			
Mean ± SD	2.6 ± 0.8	2.7 ± 0.8	
Median (IQR)	2.6 (2.1, 3.1)	2.4 (2.0, 3.1)	0.95 (MW)
*Attention score*	*n* = 63	*n* = 34	
Mean ± SD	4.6 ± 0.8	4.4 ± 0.8	
Median (IQR)	4.6 (4.2, 5.1)	4.5 (4.0, 4.9)	0.22 (MW)

SD = standard deviation; IQR = interquartile interval; T = *t*-test; MW = Mann–Whitney test.

**Table 8:** Distribution of infants according to hearing loss degree considering both the absence and presence of risk factors.

QUIT Dimensions		Hearing Loss Degree				*p*-Value (Test)
	Total †	Mild	Moderate	Severe	Profound	
**Total children with hearing loss** †	*n* = 51	*n* = 12	*n* = 20	*n* = 8	*n* = 11	
*Emotional temperament*	5.9% (3)	8.3% (1)	0.0% (0)	12.5% (1)	9.1% (1)	0.12 (F)
*Calm temperament*	3.9% (2)	0.0% (0)	0.0% (0)	12.5% (1)	9.1% (1)	
*Normal temperament*	98% (50)	100% (12)	100% (20)	87.5% (7)	100% (11)	
*Difficult temperament*	2.0% (1)	0.0% (0)	0.0% (0)	12.5% (1)	0.0% (0)	
**Hearing loss with R.F** †	*n* = 34	*n* = 9	*n* = 11	*n* = 5	*n* = 9	
*Emotional temperament*	5.9% (2)	0.0% (0)	0.0% (0)	20% (1)	11.1% (1)	
*Calm temperament*	2.9% (1)	0.0% (0)	0.0% (0)	0.0% (0)	11.1% (1)	0.12 (F)
*Normal temperament*	97.1% (33)	100% (9)	100% (11)	80% (4)	100% (9)	
*Difficult temperament*	2.9% (1)	0.0% (0)	0.0% (0)	20% (1)	0.0% (0)	
**Hearing loss without R.F** †	*n* = 17	*n* = 3	*n* = 9	*n* = 3	*n* = 2	
*Emotional temperament*	5.9% (1)	33.3% (1)	0.0% (0)	0.0% (0)	0.0% (0)	
*Calm temperament*	5.9% (1)	0.0% (0)	0.0% (0)	33.3% (1)	0.0% (0)	0.27 (F)
*Normal temperament*	100% (17)	100% (3)	100% (9)	100% (3)	100% (2)	
*Difficult temperament*	0.0% (0)	0.0% (0)	0.0% (0)	0.0% (0)	0.0% (0)	

† = some infants showed more temperament type; F = Fisher’s exact test; R.F. = risk factor.

## Data Availability

Data available on request.

## Abbreviations

The following abbreviations are used in this manuscript:

RF	Risk factors
QUIT	Italian Temperament Questionnaire
SD	Standard deviation
ABR	Auditory-evoked brainstem
